# Response to MHC‐based olfactory cues in a mate choice context in two species of darter (Percidae: Etheostoma)

**DOI:** 10.1002/ece3.11025

**Published:** 2024-02-21

**Authors:** Kara M. Million, Melissa R. Proffit, Sierra J. Reese

**Affiliations:** ^1^ Department of Biology Indiana University Bloomington Indiana USA; ^2^ Present address: Department of Ecology and Evolutionary Biology University of Tennessee Knoxville Tennessee USA; ^3^ Present address: Department of Biology University of California, Davis Davis California USA

**Keywords:** darters, Etheostoma, mate choice, MHC, olfactory cues, opposites‐attract hypothesis

## Abstract

Mate choice is hypothesized to play an important role in maintaining high diversity at major histocompatibility complex (MHC) genes in vertebrates. Many studies have revealed that females across taxa prefer the scent of males with MHC genotypes different to their own. In this study we tested the “opposites‐attract” hypothesis in two species of darter with known differences in female criteria used in mate choice: in the fantail darters (a paternal‐care species), females prefer males with visual traits related to nest guarding and egg tending, while in rainbow darters (not a paternal‐care species) female mate choice criteria are unknown. In dichotomous mate‐choice trials, we presented females of both species with the scents of conspecific males with MHC class IIb genotypes that were either similar or dissimilar to that of the focal female. We evaluated the proportion of time each female spent with each male and calculated the average strength of female preference for both species. Female fantail darters demonstrated a preference for the scent of males with similar (rather than dissimilar) MHC genotypes, but this result was not statistically significant. Rainbow darter females showed no preference for the scent of males with similar or dissimilar MHC genotypes. Our results do not support the “opposites‐attract” hypothesis in darters.

## Graphical abstract

In this study we tested the “opposites‐attract” hypothesis in two species of darter with known differences in female criteria used in mate choice: in the fantail darters (a paternal‐care species), females prefer males with visual traits related to nest guarding and egg tending, while in rainbow darters (not a paternal‐care species) female mate choice criteria are unknown. In dichotomous mate‐choice trials, we presented females of both species with the scents of conspecific males with MHC class IIb genotypes that were either similar or dissimilar to that of the focal female. Our results do not support the “opposites‐attract” hypothesis in darters.

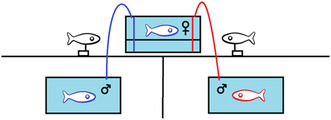

## 1 INTRODUCTION

The major histocompatibility complex (MHC) is an important component of the vertebrate immune system. MHCs are proteins on the surface of cells involved in self/non‐self recognition. MHC proteins bind peptides from foreign agents and present the peptides to activate an immune response. MHCs play a key role in recognizing pathogens and mediating the body's response to grafts (Janeway et al., 2001). Additionally, MHCs play a role in mate choice in many vertebrate taxa (Ruff et al., 2012). Vertebrates shed MHC particles in body fluids and volatiles, making MHCs a component of olfactory signals in many animals (Singer et al., 1997; Slade et al., 2016; Yamaguchi et al., 1981). The prevailing hypothesis concerning MHC‐based mate choice is that “opposites attract”: potential mates with MHC genotypes different from that of the focal individual are more attractive than potential mates with similar or identical genotypes. The proposed explanation for this hypothesis is that selecting a mate with an opposite genotype maximizes MHC diversity in the offspring. Diversity at MHC loci is thought to promote higher fitness and resistance to pathogens (Kubinak et al., 2011). In contrast to traditional mate choice theory, where females express a preference for a general phenotype, MHC‐based mate choice hypotheses focus on female preference for individuals with a genotype and phenotype specifically compatible with that of the choosing female.

Since MHC‐based mate choice was first discovered in mice (Yamazaki et al., 1976), many studies have found empirical support for the “opposites‐attract” hypothesis across most major vertebrate taxa (Dandine‐Roulland et al., 2019; Hoover et al., 2018; Huchard et al., 2013; Juola & Dearborn, 2011; Ruff et al., 2012; Setchell et al., 2010; Setchell & Huchard, 2010). One particularly well‐known study found that in humans, women preferred the scent of men who had MHC genotypes opposite to that of the focal female (Wedekind et al., 1995). Despite the support for the “opposites‐attract” hypothesis found in many studies, other studies have yielded the opposite result (focal individuals preferred the scent of individuals with similar MHC genotypes) or no conclusive evidence either way for MHC‐based mate choice (Ekblom et al., 2004; Galaverni et al., 2016; Jaworska et al., 2020; Sepil et al., 2015; Westerdahl, 2004). Which factors predict whether MHC‐based mate preferences will arise in a species remains an open question. One possibility is that MHC‐based preferences (or lack thereof) depend on what other criteria a species uses in mate choice, or on other reproductive behaviors. By comparing MHC‐based preferences across species with different reproductive behaviors and mate choice criteria, we can evaluate the extent to which these other characteristics predict the presence of MHC‐based disassortative mate choice.

Here we evaluate the “opposites‐attract” hypothesis in darters (genus *Etheostoma*), small percid freshwater fishes. More than 200 species of darter have been described to date. The group exhibits a diverse array of reproductive traits and behaviors (Page, 1985). In our study we focus on two species with different reproductive characteristics: the rainbow darter (*Etheostoma caeruleum*) and the fantail darter (*Etheostoma flabellare*). Darters are ideal for this study because we can evaluate the “opposites‐attract” hypothesis in multiple species that differ in their reproductive behaviors.

Rainbow darters display sexually dimorphic characteristics during the breeding season (Kuehne & Barbour, 1983; Page, 1983; Page & Burr, 2011). Males develop bright red and blue coloring on their bodies and fins. These colors were long thought to be attractive to females, and a possible visual signal of male quality, as the colors may be costly to maintain due to energetics and the pleiotropic effects of androgens on the immune system (Folstad & Karter, 1992). However, several recent studies have shown that the colors may be more important visual signals for male–male communication; males use the colors and patterns to distinguish heterospecific males from conspecific male rivals for mates, allowing them to reserve energy for fighting off true rivals rather than engaging with heterospecific males (Zhou et al., 2015; Zhou & Fuller, 2016). If the colors are involved in male–male communication rather than in female mate choice, the signals females of this species use in mate choice, if any, are unknown. In this species neither sex provides parental care to the offspring.

In fantail darters, breeding males do not develop bright colors, although they do darken in color and their vertical stripes become more prominent (Page & Burr, 2011). In this species males establish nests under rocks, and they engage in nest guarding and egg tending until the eggs hatch. Breeding males develop “egg mimics,” amber‐colored knobs, on their dorsal fins (Page & Bart, 1989). Behavioral studies show that females of this species prefer males possessing egg mimics to males without them and that females prefer males that already have egg clutches to males without them (Knapp & Sargent, 1989). It is hypothesized that these visual signals indicate the quality of a male as a caregiver to the eggs. In nest‐guarding species of darter, males will sometimes engage in allopaternal care in which they guard eggs that are not their own offspring, most likely because doing so will increase their opportunities to mate (Porter et al., 2002). Although the visual components of mate choice in darters have been well‐studied, little is known about the potential role olfactory cues play in mate choice in darters. Several studies have demonstrated that darters respond to olfactory chemical cues such as alarm cues (Commens & Mathis, 1999; Haney et al., 2001; Smith, 1979), and our results from a previous study suggest that our focal species respond to olfactory cues in a mate choice context (unpublished data).

The purpose of our study was to evaluate the MHC‐mediated disassortative mate choice hypothesis in these two species of darter to determine whether females of either species demonstrate preferences for the scent of males with MHC genotypes dissimilar to their own. We further sought to determine whether different mate choice criteria and reproductive behaviors between closely related species can lead to different MHC‐based preferences. If the “opposites attract” hypothesis is true for either species, we expected females in reproductive condition to prefer the scent of males with dissimilar MHC genotypes to that of the focal individual over the scent of males with similar MHC genotypes.

## 2 METHODS

### 2.1 Subject collection

Individuals of the rainbow darter (*Etheostoma caeruleum*) and the fantail darter (*Etheostoma flabellare*) were collected with a seine net from Clear Creek, Bloomington, Indiana, Monroe County (39.12118432195659, −86.53886585996088) on April 6, 2019. We identified individuals to species based on morphological characteristics (Page & Burr, 2011; Simon, 2011). Individuals were sexed based on breeding characteristics. In both species, we identified females as gravid by the presence of distended abdomens. We identified male rainbow darters in breeding condition by nuptial coloration, and male fantail darters in breeding condition by darkened body color and the presence of egg mimics on the first dorsal fin (Kuehne & Barbour, 1983; Page, 1983). Individuals were transported to Indiana University in aerated, covered buckets in an air‐conditioned vehicle.

### 2.2 Care and housing

Individuals were housed separately by sex and species. Males and females of the same species were housed such that they were unable to see conspecifics of the opposite sex. We housed individuals in 75.7‐L tanks (76.2 × 30.48 × 30.48 cm) equipped with power filters and air pumps mounted with large air stones. River rock aquarium gravel was provided as substrate. Small terra cotta pots were provided as hides. We conditioned the tank water with Amquel Plus and performed 20% water changes once per week. Individuals were fed frozen brine shrimp and bloodworms once per day, as much as could be consumed in 5 min.

### 2.3 Fin clipping and VIE tagging

Individuals were fasted overnight ahead of the fin clipping and tagging procedures. Individuals were anesthetized with MS‐222 (25 mg/L) until gill movement was slow and steady and the subject did not respond to touch. Paper towels moistened with conditioned water were placed on the gill covers to maintain the subject's breathing during the procedure. Each subject was measured for standard length (tip of the nose to the caudal peduncle). A small (3 mm^2^) piece of fin tissue was removed from the anal fin of each subject. Iodine was used to sterilize the fin clip site during the procedure. Each individual was injected with three visible implant elastomer (VIE) tags (Northwest Marine Technology), one anterior to the first dorsal fin, one between the first and second dorsal fin, and one behind the second dorsal fin. Each VIE tag combination was a unique color pattern used to identify the individual. The total procedure from start to finish took approximately 3 min. Individuals were placed in conditioned water and allowed to recover from the anesthesia. Recovery was pronounced when the subject resumed normal swimming and breathing. All individuals were monitored over the following 24 h for full recovery. All fin tissue regenerated within 3 weeks, and all VIE tag wounds healed within 1 week with no sign of infection or long‐term effects. Behavioral trials were not performed until all individuals had fully healed and recovered from fin clipping and VIE tagging. Fin clips were frozen and stored for genotyping.

### 2.4 MHC genotyping

All individuals were genotyped at a 164 base pair portion of the MHC class IIb gene (exon 2) according to deep‐sequencing methods developed for fish (Lighten et al., 2014). We used custom primers and protocols we had developed for previous research on darters (Million & Lively, 2022). Our prior research indicated the presence of up to 5 MHC IIb loci in both our species of interest, meaning an individual genotype could contain 1 to 10 alleles. The number of alleles was recorded for each genotype as well as the identity of each allele in each genotype.

### 2.5 Breeding conditions in the lab

To maintain the breeding condition of all individuals in the lab during the trials, we manipulated ambient light and temperature to mimic the conditions of the natural breeding season. We kept the day length cycle at 14 L:10D and maintained the ambient room temperature at 28°C. We assessed the success of our efforts by observing female gravidity and male nuptial characteristics as described above.

### 2.6 Construction of motorized visual stimuli

**FIGURE 1:**
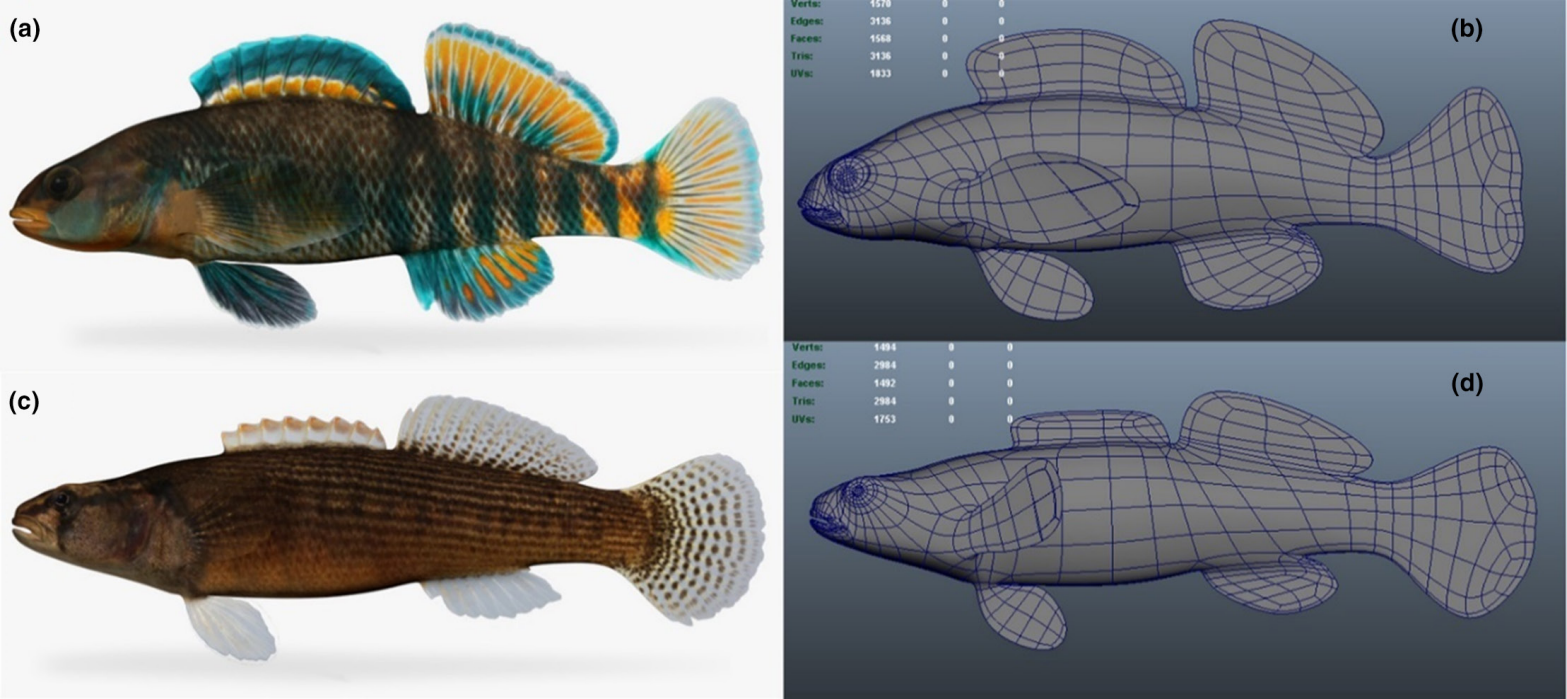
3D models obtained from Turbosquid for construction of motorized male visual stimuli (a and b: rainbow darter male; c and d: fantail darter male).

**FIGURE 2:**
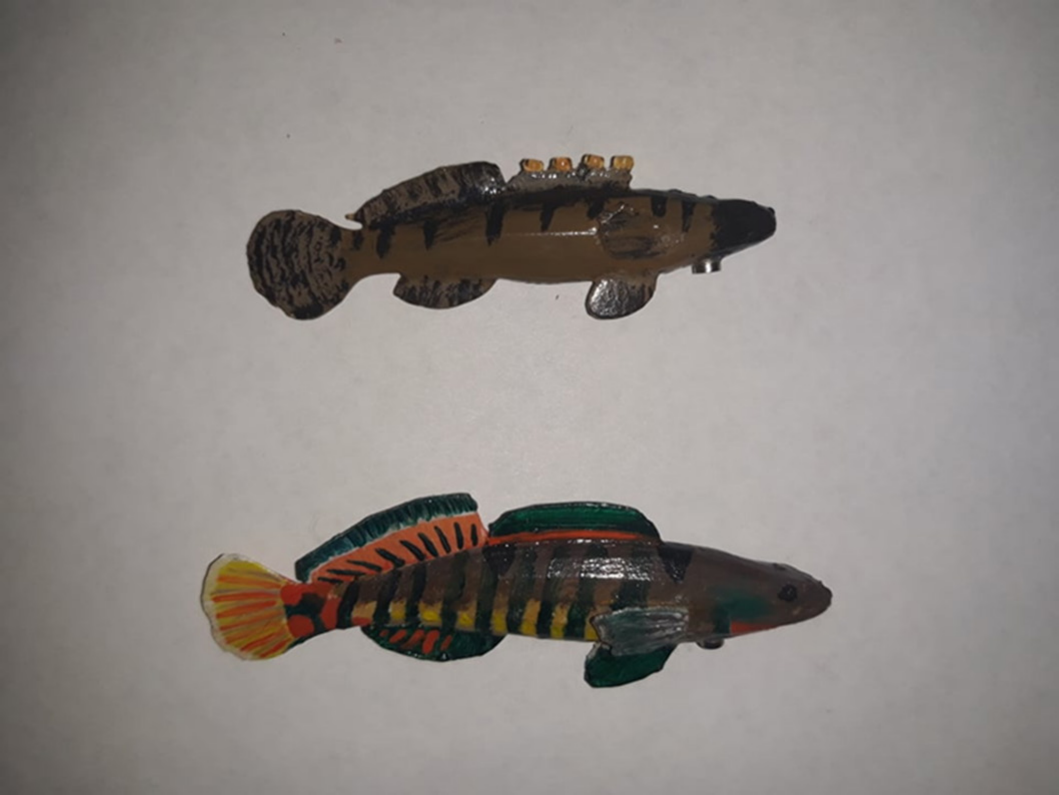
Male models used as visual stimuli for choice trials. Top: fantail darter male. Bottom: rainbow darter male.

To control for possible differences in visual and behavioral cues between live males, we constructed motorized visual stimuli with which to pair the olfactory cues during the trials. These models allowed us to present identical visual stimuli to the focal females and provide a visual context in which to interpret the olfactory stimuli. Motorized, painted models have been successfully used by other researchers for dichotomous choice trials in darters (Williams et al., 2013; Williams & Mendelson, 2010). 3D models of a male fantail darter and a male rainbow darter were purchased from the website TurboSquid (model artist: “adicaza”) (Figure 1). Files were modified for compatibility with a 3D printer. Life‐sized male models were 3D printed using a Formlabs Form 2 printer and Formlabs SG dental resin. 3D printing was performed in the Lennon lab at Indiana University. High‐resolution images of a male fantail darter and a male rainbow darter were obtained from the North American Native Fishes Association photo gallery, and color palettes were developed from colors isolated from the images. Models were painted with colors and patterns resembling a male of each species in breeding condition (Figure 2). Models of the same species were painted to be visually identical. Small glass amber‐colored beads (2 mm outside diameter) were glued to the first dorsal fin of the fantail darter models to resemble egg mimics. Models were mounted onto stepper motors using magnets and transparent plexiglass platforms. Stepper motors were attached to microcontrollers, which in turn were connected to an Arduino Uno REV3 unit. Model movements were programmed using the software Arduino IDE 1. The Arduino unit was programmed so that the models made synchronized movements that resembled the courtship displays of live breeding males. Two visually identical models of each species were created.

### 2.7 Experimental setup

A 37.9‐L tank (50.8 cm″ × 25.4 cm × 30.5) was set up in a location where the subject would have no visual contact with other individuals during the trial (Figure 1). The focal tank was surrounded with sheets of panda paper (Vivasun) to block external light and visual stimuli. The bottom of the tank was blacked out using matte black aquarium decal material. The tank was separated into three zones of preference, marked by narrow white lines. The neutral zone was centered in the middle and took up 80% of the tank bottom surface area. Each preference zone on the left and right took up 10% of the tank bottom surface area. Ambient temperature in the trial area matched that of the housing area. The tank was lit using ambient fluorescent light and a Nicrew ClassicLED Plus full‐spectrum aquarium light mounted above the tank. Two 37.9‐L tanks were placed underneath the focal tank, completely out of the visual range of the focal subject. All tanks were partially filled with conditioned, aerated water (3/4 full for the focal tank, ½ full for each olfactory stimulus tank). At the conclusion of each trial, all tanks were emptied, scrubbed with 95% ethanol, allowed to air dry, and refilled with the same volume of fresh water. An AKASO EK7000 Pro 4K Action Camera was mounted near the tank to record all trials. All motorized and electronic devices were connected to a remote control so that they could be turned on and off without the observer in the room during a trial. The trials were monitored outside the room in real time using a cell phone remotely connected to the camera.

We pre‐determined that if a participant showed signs of severe stress (gasping, attempting to jump out of the tank) at any point during a trial, then the trial would have been immediately terminated and the subject returned to housing. However, during these trials no individuals displayed signs of extreme stress, so we did not terminate any of the trials. No individual participated in more than one trial per 24‐h period, either as a subject or as a stimulus individual. Submersible pumps (Homasy 300 L/H, 4 W) were placed into each olfactory stimulus tank. The pumps were connected to the focal tank via clear food grade vinyl tubing (7.9 mm ID, 11.11 mm OD). The pumps were primed with clean conditioned water before and after every trial. Plastic clamps were used to control the water flow and to ensure that the water flowed into the focal tank at the same rate on each side. This was visually confirmed prior to the start of every trial.

### 2.8 MHC‐based choice trials

**FIGURE 3:**
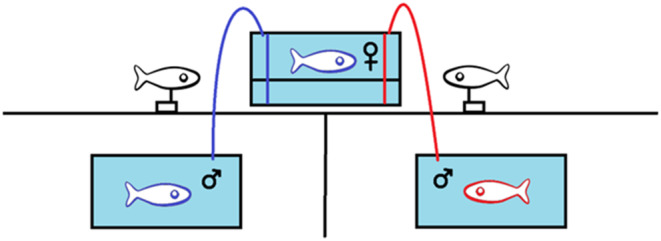
Experimental setup for choice trials. The top tank is the focal tank containing the focal individual. Identical motorized male models are positioned on either side of the focal tank. Olfactory stimulus tanks are under the focal tank out of the visual range of the focal individual. One live stimulus male is contained in each stimulus tank. Water is being gently pumped into the focal tank on either side. The stimulus male on the left has a genotype similar to that of the focal female as indicated by the matching colors. The stimulus male on the right has a genotype dissimilar to that of the focal female, as indicated by the mismatched colors.

We used dichotomous choice trials to assess the response of females of each species to MHC‐based olfactory cues from conspecific males (Figure 3). The method is standard for evaluating mate choice preferences in darters (Mattson et al., 2020; Williams et al., 2013). For each trial, to control for visual and behavioral differences between the two live males, two visually identical male models of the same species as the focal female were placed on either side of the focal tank and mounted on stepper motors. Before the start of the trial, blinds were used to block the view outside the tank of the focal female during acclimation. One focal female was acclimated to the focal tank for a minimum of 10 min. A female was considered acclimated when she initiated normal free swimming around all sides of the tank and exhibited normal breathing patterns. One live stimulus male was acclimated to each of the olfactory stimulus tanks out of view of the focal female for 10 min, the same amount of time that the focal female was acclimated to the focal tank.

After the acclimation period, the blinds were removed, and the focal female was allowed to explore the tank and visually inspect each model. The trial began after the female had crossed into both preference zones, seen both models, and returned to the neutral zone. A trial did not progress if the focal individual did not cross both sides and return to the middle. At the start of the trial, both male models were turned on to provide identical visual displays for the female, and water was pumped into the focal tank on either side of each of the stimulus tanks. During each trial the focal female was presented with the scent of a male with an MHC genotype similar (differing by no more than one allele) or identical to her own on one side, and one male with an MHC genotype completely dissimilar to her own (none of his alleles matched hers) on the other side. To control for heterozygosity, we matched stimulus males so that they either had the same number of alleles in their genotypes or only differed in number by one. Due to small sample sizes, some females participated in more than one trial. In these cases, a female was always presented with a completely novel pair of stimulus males. The trial period lasted for 10 minutes, and the entire trial was observed outside the room by camera and was recorded. To control for potential side bias in the focal females, we switched the sides on which the similar and dissimilar male scents were presented to the focal female between each trial, and we switched the sides of the two models between each trial. Sample sizes for the trials were *N* = 4 for rainbow darters and *N* = 5 for fantail darters. Total number of trials run were *N* = 9 for rainbow darters and *N* = 8 for fantail darters.

### 2.9 Trial analyses

Videos were analyzed using the software Boris (Friard & Gamba, 2016). We constructed an ethogram in the software to use during analysis. All videos were scored for the amount of time the focal female spent in each of the three zones of the focal tank. If during a trial a female spent more than 80% of her time in the neutral zone, we concluded that she had not interacted enough with the stimuli for us to score her preferences, and that trial was set aside from further analysis.

A strength of preference (SOP) equation was used to evaluate the responses to the stimuli. The equation is SOP = (S – D)/(S + D), where S = the time spent in the preference zone with the male with a similar MHC genotype and D = the time spent in the preference zone with the male with a dissimilar MHC genotype. The values obtained range from a minimum of −1 (strong preference for dissimilar genotype) to a maximum of 1 (strong preference for a similar genotype). A value at or near 0 indicates no strong preference for either genotype. Further data analysis was conducted using the software R version 4.1.0 (R Development Core Team, 2018). Mean SOP between the species was compared using a Wilcoxon rank‐sum test. Proportion of time spent with the male with the similar MHC genotype (as opposed to the male with the dissimilar MHC genotype) was tested against the null hypothesis that the females spent equal time with both males using a one‐sample Wilcoxon test. Power analyses on the data were conducted using the software G*Power.

## 3 RESULTS

Eight trials were run on fantail darter females, and nine trials were run on rainbow darter females. Three trials for the fantail darters were set aside from analysis because the focal females spent more than 80% of their time in the neutral zone. In one fantail darter trial, the identity of one of the stimulus males (the “similar” male) was mislabeled. Because we could not verify the identity of this male, this trial was also set aside from analysis. Thus 4 trials for the fantail darters were included in the final analysis. The final sample sizes were *N* = 4 for rainbow darters and *N* = 3 for fantail darters (one individual participated in two trials).

**FIGURE 4:**
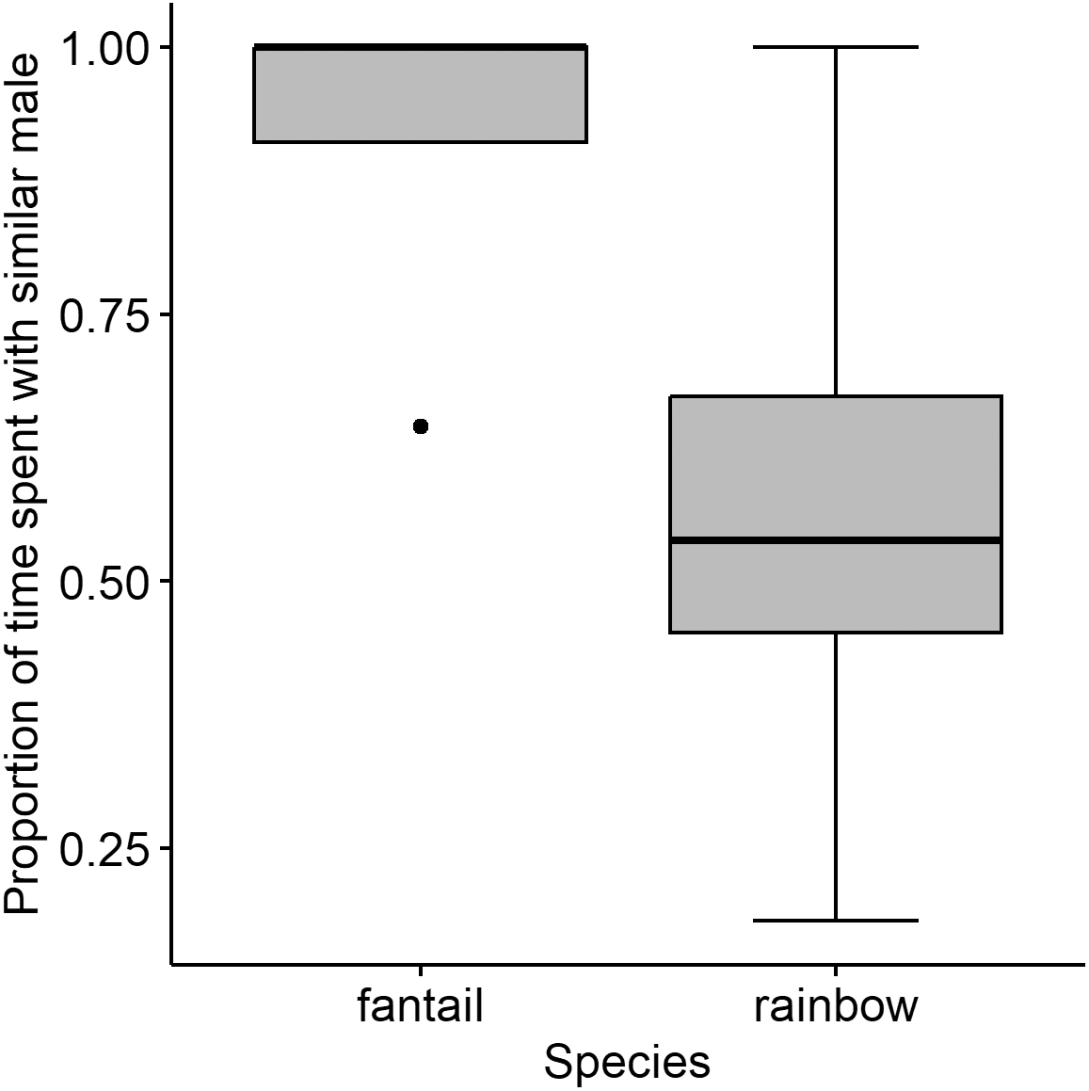
Average proportion of time spent with the scent of males with genotypes in females of both species.

Fantail darter females spent more time associating with the scent of males with a similar genotype to that of their own (mean proportion of time with similar genotype = 0.91, SE ± 0.09), but this result was not statistically significant (*V* = 10, p = .09) (Figure 4). Rainbow darter females spent approximately equal time associating with the scent of males with similar and dissimilar genotypes to their own (mean proportion of time with similar genotype = 0.56, SE ± 0.08, V = 29, *p* = .50).

Average strength of preference (SOP) for rainbow darter females was 0.15 (SE ± 0.15), and average SOP for fantail darter females was 0.82 (SE ± 0.18). Although SOP was higher in fantail darter females, this difference was not statistically significant (Wilcoxon test, *p* = .06).

Between the two preference zones, each fantail darter female spent more than 60% of her time with the scent of the male with a similar genotype to her own. In rainbow darter females, individual proportions of time spent in each zone varied; in four trials females spent more time with the scent of the similar genotype, while in three trials females spent more time with the scent of the dissimilar genotype, and in two trials females spent nearly equal time with each stimulus.

The calculated effect size for the SOP means was 3.918, and the effect sizes for the fantail darter and rainbow darter mean proportions of time spent with the similar male were 0.945 and 0.257, respectively. Given the effect sizes, an alpha value of 0.05, and the sample sizes, the post hoc power analyses indicated that we achieved enough statistical power for the SOP means analysis, but not for the analyses of the mean proportions for each species. The sample size analyses indicated that, given an effect size of 0.8 and an alpha value of 0.05, the required sample size to achieve statistical power of 80% is *N* = 30 per group for the SOP analysis, and *N* = 17 for the proportion analyses.

## 4 DISCUSSION

There were three possible conclusions we could have reached in our study: (1) there is disassortative mate choice for dissimilar MHC genotypes in female darters, (2) there is assortative mate choice for similar MHC genotypes in female darters, and (3) there is no MHC‐based mate choice in female darters. Our results for fantail darter females were the opposite of what we would have predicted in the case of disassortative MHC‐based mate choice in this species. All our focal females spent more time associating with the scent of males with MHC genotypes similar to that of the focal female. Therefore, we found no evidence for disassortative mate choice in fantail darter females. The remaining possibilities are assortative mate choice for similar MHC genotypes, or no MHC‐based mate choice. While our results suggest that there may be assortative MHC‐based mate choice in females of this species, our ability to draw a definitive conclusion is limited by a small sample size. We had to discard many trials on the fantail darters because some females did not interact enough with the models. This issue has been reported by other researchers attempting to run dichotomous choice trials in this species (Tamra Mendelson and Rachel Moran, personal anecdotes). Another difficulty with interpreting the results is that some of the females participated in more than one trial, which means the trials are not all completely independent of one another. We attempted to mitigate this issue by presenting the females with a completely independent pair of males for each trial, but this did not completely eliminate the problem. However, the results for the re‐used female were identical between the two trials; she spent all her time associating with the similar male, and no time associating with the dissimilar male even though the side on which the similar male appeared was switched between trials.

Despite the difficulties of interpreting our results, these preliminary data allowed us to conduct power analyses and to calculate the ideal sample sizes for this type of study. These analyses give us a promising path forward for follow‐up studies on MHC‐based mate choice in darters.

The “opposites‐attract” hypothesis for MHC‐based mate choice is intuitively appealing because it is based on the idea that mating with opposite MHC genotypes produces more genetic diversity in the offspring, which would confer a fitness advantage to the progeny (Kubinak et al., 2011). It is less clear why females would prefer to mate with males with similar MHC genotypes, especially since doing so may reduce diversity in offspring, which is thought to confer a fitness disadvantage. Nevertheless, some studies have found evidence for assortative mate choice in several taxa (Bos et al., 2009; Sin et al., 2015). Offspring with highly divergent or extremely diverse MHC genotypes may be at higher risk of autoimmune disorders or other fitness costs (Ilmonen et al., 2007; Kalbe et al., 2012; Kubinak et al., 2011; Wegner et al., 2003, 2004). Disassortative MHC‐based mating may break up genotype combinations that are locally adapted to pathogens that occur in the individuals' environment. Follow‐up studies in fantail darters could explore the reasons fantail darter females appear to prefer the scent of males with similar MHC genotypes to their own.

We found no evidence for a preference for the scent of males with dissimilar MHC IIb genotypes in females of either of our focal species. Rainbow darter females were split in their responses to the stimuli, with some spending more time with the scent of the MHC‐similar males, some spending more time with the scent of the MHC‐dissimilar males, and some spending equal time associating with both scents. Although there is evidence for individual variation in mate‐choice preferences in some vertebrates (Bleay & Sinervo, 2007; Houde, 1988; Ronald et al., 2018; Zietsch et al., 2011), and evidence that individual mate‐choice preferences can be influenced by individual differences in sensory processing abilities (Ronald et al., 2018; Santos et al., 2018), we cannot rule out the possibility that our females were not making their choices based on MHC genotype at all and were instead making choices based on a different set of criteria. Until we run additional experiments to distinguish between these two explanations, we conclude that our study did not find evidence of MHC‐based mate choice in rainbow darters. It would be worthwhile to conduct further choice trials to determine whether rainbow darter females have mate preferences based on other MHC genes (such as class I genes rather than class II), or based on other criteria entirely. Additionally, follow‐up studies could test for individual variation in mate‐choice‐related preferences in rainbow darter females. Because male nuptial coloration plays a larger role in male–male signaling in this species than in female mate choice and we found no support for olfactory‐based disassortative mating preferences, the factors that influence mating preferences in rainbow darter females remains an open question.

Many of our *E. flabellare* females were excluded from this study because they did not spend enough time engaging with the stimuli. Other researchers have reported difficulty running behavioral studies in darters in this subgenus, although the reasons are unknown. In our case, the male models we made for this species were based on a reference photo of an *E. flabellare* subspecies, *E. flabellare humerale*. The dark bar patterning on this subspecies may differ from that of the males in our focal population (*E. flabellare flabellare*), and if females can distinguish the differences, they may have had difficulty recognizing the model as a conspecific male. This may have impacted our results. Future studies using male visual stimuli should use reference photos taken of males from the actual study population.

In summary, we observed a difference between our focal species in their preferences, although this difference was not statistically significant. Fantail darter females preferred MHC‐similar male odors, while Rainbow Darter females showed no definitive preference either way. The average strength of preference was higher in fantail darters than in rainbow darters, although the observed difference was not statistically significant. Prior studies have indicated that female mate choice plays a larger role in darters from the subgenus *Catonotus* (such as the fantail darter) than in darters from the subgenus *Oligocephalus* (such as the rainbow darter) (Fuller, 2003). These two groups of fishes have different reproductive behaviors and visual criteria for mate choice, as female *Catonotus* darters have visual preferences for males with traits related to paternal care (Knapp & Sargent, 1989). The differences in olfactory preferences could be a result of different reproductive strategies and selection pressures related to the presence or absence of paternal care. Male‐only parental care has evolved multiple times independently in darters (Kelly et al., 2012). Future studies could test MHC‐based mate choice preferences in multiple taxa with and without paternal care to determine whether assortative MHC‐based mate choice is associated with paternal care species.

One factor that deserves future consideration is that male mate choice has been shown to play a role in sexual selection and reproductive isolation in darters (Zhou et al., 2015; Zhou & Fuller, 2016). Research indicates that male preference for conspecific visual traits reinforces reproductive isolation because it allows the males to avoid mating with heterospecific females (Mendelson, 2003b; Moran et al., 2017). Males of several species of darter have demonstrated preferences for conspecific colors and patterns over heterospecific colors and patterns (Mendelson, 2003a, 2012; O'Rourke & Mendelson, 2010; Zhou et al., 2015). Male rainbow darters have also demonstrated a preference for larger conspecific females (Soudry et al., 2020). Additionally, male MHC‐based mate choice has been found in a sex role‐reversed fish (Roth et al., 2014). If male mate choice plays a significant role in the evolution and diversification of darters, it would be worthwhile to test for MHC‐based odor preferences in males in future studies.

In conclusion, our study finds no evidence that MHC‐based disassortative mate choice maintains MHC class II genetic diversity in darters. In fact, in some species of darter MHC‐based assortative mate choice may be selecting against diversity at MHC genes. Although mate choice may play a role in maintaining MHC diversity in other species, in darters the mechanisms are more likely related to other factors, such as pathogen‐mediated selection pressures, than to reproductive behaviors. However, we strongly caution against drawing strong conclusions from the results of this study due to the small sample size. Further experiments are needed with larger sample sizes to confirm these results.

## AUTHOR CONTRIBUTIONS

**Kara M. Million:** Conceptualization (lead); data curation (lead); formal analysis (lead); funding acquisition (lead); investigation (lead); methodology (lead); resources (equal); supervision (lead); visualization (lead); writing – original draft (lead); writing – review and editing (lead). **Melissa R. Proffit:** Conceptualization (equal); data curation (equal); formal analysis (supporting); methodology (equal); supervision (equal); visualization (supporting); writing – original draft (supporting); writing – review and editing (supporting). **Sierra J. Reese:** Conceptualization (supporting); data curation (supporting); formal analysis (supporting); investigation (supporting); methodology (supporting); writing – original draft (supporting); writing – review and editing (supporting).

## FUNDING INFORMATION

This work was supported by the National Institute of Health (Common Themes in Reproductive Diversity training grant T32 HD049336 to KMM), The National Science Foundation (CISAB Research Experience for Undergraduates grant number 1460949 to SJR), The American Society of Naturalists, The Society for the Study of Evolution, Sigma Xi, Indiana Academy of Science, and the American Society of Ichthyologists and Herpetologists.

## CONFLICT OF INTEREST STATEMENT

The authors declare no competing interests.

## Supporting information


Data S1

Table S1

## Data Availability

Data are available at https://doi.org/10.5061/dryad.t76hdr82h.
