# Supplementary material for: Response to MHC‐based olfactory cues in a mate choice context in two species of darter (Percidae: Etheostoma)
Source: Ecol Evol. 2024 Feb 21;14(2):e11025. doi: 10.1002/ece3.11025 (PMC10880076; doi:10.1002/ece3.11025)
Supplement: Supplementary file 2 — Table S1 [file ECE3-14-e11025-s001.docx]

Table S1: Ethogram used in the software Boris to score darter behavioral trials. Computer key was pressed and held down for the entire duration of the behavior until the focal individual crossed into one of the other zones.

| **Computer key** |  | **Behavior recorded** |
| --- | --- | --- |
| L |  | Crossed into left zone |
| R |  | Crossed into right zone |
| N |  | Crossed into neutral zone |
